# Mixed state behavior of Hermitian and non-Hermitian topological models with extended couplings

**DOI:** 10.1038/s41598-023-33449-9

**Published:** 2023-04-20

**Authors:** Y. R. Kartik, Sujit Sarkar

**Affiliations:** 1 Theoretical Sciences Division, Poornaprajna Institute of Scientific Research, Bidalur, Bangalore, 562164 India; 2 Graduate Studies, Manipal Academy of Higher Education, Madhava Nagar, Manipal, 576104 India

**Keywords:** Topological insulators, Theoretical physics

## Abstract

Geometric phase is an important tool to define the topology of the Hermitian and non-Hermitian systems. Besides, the range of coupling plays an important role in realizing higher topological indices and transition among them. With a motivation to understand the geometric phases for mixed states, we discuss finite temperature analysis of Hermitian and non-Hermitian topological models with extended range of couplings. To understand the geometric phases for the mixed states, we use Uhlmann phase and discuss the merit-limitation with respect extended range couplings. We extend the finite temperature analysis to non-Hermitian models and define topological invariant for different ranges of coupling. We include the non-Hermitian skin effect, and provide the derivation of topological invariant in the generalized Brillouin zone and their mixed state behavior also. We also adopt mixed geometric phases through interferometric approach, and discuss the geometric phases of extended-range (Hermitian and non-Hermitian) models at finite temperature.

## Introduction

Topological state of matter is the area of huge interest both from the theoretical and experimental aspects^1–4^. These states lack the conventional local order parameter to characterize the phase transition and can not be apprehended by the traditional Landau theory of symmetry breaking^5,6^. By emerging as a suitable platform for the realization of exotic particles other than the fermions and bossons, the area attracted a great attention^7,8^. A recent addition is the non-Hermitian topological phases. In non-Hermitian systems, the energy spectrum becomes complex and the band structure may change drastically based on the type of non-Hermitian factor^9–11^. Non-Hermitian systems are believed to be a better platform to understand the loss and gain in the areas of optical and open quantum systems^12–14^. However, majority of the Hermitian and non-Hermitian topological transitions have been recognized as the second order quantum transitions at zero temperature, and can be explained from the perspective of pure quantum states^15–17^.

In the pure states, Berry phase is an interesting phenomenon, which is a quantum phase and remains purely geometrical by excluding the dynamical contribution of the time evolution of states^18–20^. This can be called topological, if the phase is invariant under the path deformation of the evolving quantum state. For a quantum system, which is in contact with the environment, the evolution is affected and results in the production of noise in the form of thermal fluctuations. The quantum states are sensitive to these thermal noises and can become undefined at these instances. This creates a necessity to define topology at finite temperature and gives the idea of mixed quantum states^20–22^. The invariance of the geometrical phase during path deformation of mixed state evolution defines the topological invariant at finite temperature in the form of Uhlmann phase^23–31^. Recently there are also efforts to explain the finite temperature behavior based on ensemble geometric phases^32,33^ and Wilson loop^27^ approach for different topological models.

The topological states are protected by certain discrete symmetries and can be classified based on 10-fold symmetry classification. Different symmetry classes possess different physical properties and violation in symmetries reflect in the physical properties of the system^2,3^. One of the symmetry class called parity-time (${\mathcal{P}\mathcal{T}}$) symmetry has efficiently explained the physical properties of non-Hermitian systems^34–38^. However, the non-Hermitian systems possess a different periodic table for symmetry classifications which decides the physical property of the system^39^. These physical properties are maintained at zero temperature and the introduction of temperature destroys topological properties of the system. This is a temperature driven transition, where the zero temperature and high temperature represent the pure state and maximally mixed states respectively. The phase transition occurs at critical temperature $T_c$, bellow which the initial topological properties of the systems are preserved^31^.

Topological phases are characterized based on the numbers called topological invariants, which are in correspondence with the number of localized edge modes^40,41^. It is possible to generate higher winding numbers (WNs) either by increasing the number of coupling sites (static method)^42^ or by periodic driving (dynamical method)^43^. However, this observation is true for BDI and AIII symmetry classes and holds good only for certain limit. The breaking of symmetry may violate the above property^44^. With the infinite neighbor coupling, one can obtain long-range models which are interesting from the perspective of massive edge modes and breaking of Lorentz invariance^44–50^. The long-range models have been experimentally realized in trapped ions^51–54^, atom coupled to multi- mode cavities^55^, magnetic impurities^56,57^ and simulated circuits^58^. They have a advantage of suppressing the finite sized effect over to short-range models^59^. The massive edge modes are found to be an effective qubits in topological computations^48^. On the other hand, non-Hermitian system exhibit sensitivity towards the boundary conditions, and there exists non-Hermitian skin effect which creates an extra localization of eigen states in the open boundary condition.

**Motivation** Dealing the topological state of matter at finite temperature as a condition of mixed state is an interesting area from the perspective of both theory and experimentation. Here our motivation is three-folded.

- So far, there are many efforts to understand the behavior of geometric phase for mixed states through different approaches. In the work Ref.^23^, Viyuela et al., has shown that the Uhlmann phase can be an efficient tool to measure topology at finite temperature. The work of Zang et al. (Ref.^27^), gives the signature of analyzing the possible higher WNs through the mixed state approach (in a four band model). The authors of Refs.^31,60^ shows the limitations in Uhlmann phase, and explore interferometric phase as a better geometric phase at finite temperature. However, the question about measurement of higher WNs at finite temperature still remains unanswered. In a recent work, we have observed the staircase of topological transitions in 1D extended-range models^42^. The extended-range of coupling creates higher WNs, and the model reduces to short-range with the increase in the decay parameter. The staircase of transition occurs between even-even (odd-odd) WNs for even (odd) number of interacting neighbors. Here, we are motivated to understand the possibility to define higher WNs and transitions among them at finite temperature.
- Mixed state approach can be an efficient tool to understand the geometry of non-Hermitian systems, where the system is connected to a thermal bath. Here we consider the non-Hermitian model (which exhibits local imbalance in the hopping amplitudes) and try to explain the topological invariant at finite temperature. Here we also extend our interest to understand the interplay of topology and long-range effects at finite temperature.
- Some topological systems exhibit localization at criticality^40,61–71^, and hence there occurs a necessity of defining topology at gapless phases. In our work (Refs.^72,73^), we have observed a transition among critical regions across a multi-critical point. This is an interesting behavior which can be observed in models extended-range coupling. This finding signals that, bulk gap is not a necessary condition to perform bulk-boundary correspondence^40,62^. However, here we extend our studies towards topological invariant for mixed states at gapless condition and thereby to understand the relation between localized edge modes and topology of criticality at finite temperature.

Here we consider a topological model connected to a thermal bath at temperature *T* and can be effectively expressed through Gibb’s ensemble. In order to explain the topological invariant at finite temperature, we use two different approaches called Uhlmann and interferometric phases.

## Model Hamiltonian and properties

We consider a generalized two band model for a one dimensional system, which can reflect both Hermitian and non-Hermitian properties based on the system parameter^74^.

1 $$\begin{aligned} H(k)=\chi _x(k)\sigma _x+\chi _y(k)\sigma _y+\chi _z(k)\sigma _z, \end{aligned}$$

where $\chi _{x,y,z}$ are the pseudo-spin vectors as a function of quasi-momentum *k* and $\sigma _{x,y,z}$ are the Pauli spin matrices. Under the Hermitian condition, the above Hamiltonian behaves $H(k)=H(k)^{\dagger }$ and under non-Hermitian condition, it is $H(k)\ne H(k)^{\dagger }$. Because of the Hermiticity, the left and right eigenstate are defined as,

2 $$\begin{aligned} H(k)|\psi _{k\pm }\rangle= & {} E_{\pm }(k)|\varphi _{k\pm }\rangle ,\nonumber \\ H(k)^{\dagger }|\psi _{k\pm }\rangle= & {} E_{\pm }^*(k)|\varphi _{k\pm }\rangle . \end{aligned}$$

with $E_{\pm }(k)=\pm \sqrt{(\chi _x(k))^2+(\chi _y(k))^2+(\chi _z(k))^2}$. For the Hermitian condition, we work on the orthonormal basis, i.e.,$|\psi _{k\pm }\rangle =|\varphi _{k\pm }\rangle$ and $E_{\pm }(k)=E_{\pm }^*(k)$. For the non-Hermitian systems, we consider biorthonormal basis, i.e., $\langle \psi _{k\nu }|\varphi _{k\mu }\rangle =\delta _{\nu ,\mu }$ and $|\varphi _{k\mu }\rangle \langle \psi _{k\mu }|=1$. Here $\nu$ and $\mu$ represent the state ‘+ ’and ‘-’respectively. By normalizing with factor $\sqrt{\langle \psi _{k,\pm }|\varphi _{k,\pm }\rangle }$, we get

3 $$\begin{aligned} |\psi _{k,\pm }\rangle= & {} \frac{1}{\sqrt{2E_{\pm }(E_{\pm }-\chi _z)}}(\chi _x-i\chi _yE_{\pm }-\chi _z)^T,\nonumber \\ \langle \varphi _{k,\pm }|= & {} \frac{1}{\sqrt{2E_{\pm }(E_{\pm }-\chi _z)}}(\chi _x+i\chi _yE_{\pm }-\chi _z), \end{aligned}$$

with *T* as transpose operator. The geometric phase due to the adiabatic evolution is given by,

4 $$\begin{aligned} \gamma =\oint \frac{\langle \psi _{k,+}|i\partial _k|\varphi _{k,+}\rangle }{\langle \psi _{k,+}|\varphi _{k,+}\rangle }dk. \end{aligned}$$

which yield the quantized values for the gapped topological phases^19^. For Hermitian systems, the azimuthal angle ($\phi$) is real and for non-Hermitian it is complex. This behavior is because of the pseudo-spin vectors, which are real for Hermitian systems and complex (at least one component) for non-Hermitian system respectively^75^, i.e., $\phi =\phi _{real}(k)+\phi _{imaginary}(k)$. Here the real and imaginary parts contribute to the argument and amplitude respectively. The term $\phi _{imaginary}(k)$ do not creates any effect on the topology of the system. The non-Hermiticity creates two exceptional points instead of single Dirac (gap closing) point. The curvature function around the exceptional points is given by $F1(k,\textbf{M})$ and $F2(k,\textbf{M})$ respectively and the winding number is given by^74,75^,

5 $$\begin{aligned} W=\left( \frac{1}{2\pi }\right) \left( \frac{\oint F1(k,\textbf{M})dk+\oint F2(k,\textbf{M})dk}{2}\right) . \end{aligned}$$

where $F1(k,\textbf{M})=\partial _k\tan ^{-1}\left( \frac{\chi _y^{re}(k)+\chi _x^{im}(k)}{\chi _x^{re}(k)+\chi _y^{im}(k)}\right)$ and $F2(k,\textbf{M})=\partial _k\tan ^{-1}\left( \frac{\chi _y^{re}(k)-\chi _x^{im}(k)}{\chi _x^{re}(k)+\chi _y^{im}(k)}\right)$ respectively. (For the detailed derivation, refer the “Method” section). As our model contains imaginary component only in $\chi _y$ term, the exceptional points are located at $(0, \chi _y^{im})$ and $(0, -\chi _y^{im})$ under the criticality condition $\chi _x^2+\chi _y^2=0$. Due to the non-Hermiticity, each exceptional point induces its own origin of pseudo-spin space and corresponding winding vectors (WVs). Thus we work on extended winding vectors (EWVs) to understand the non-Hermitian effect in the parameter space. i.e.,

6 $$\begin{aligned} \chi _x(extended)= & {} \chi _{Ex}=\chi _x^{re}(k)\pm \chi _y^{im}(k),\nonumber \\ \chi _y(extended)= & {} \chi _{Ey}=\chi _y^{re}(k), \end{aligned}$$

which correspond to parameter space of $F_1(k,\textbf{M})$ and $F_2(k,\textbf{M})$ respectively (For detailed study, please refer “Method” section).

Here we consider 1D Su-Schrieffer-Heeger (SSH) model in both Hermitian^76^ and non-Hermitian versions^74,75,77^. The model has its importance in understanding of different experimental realizations related to topology^78–80^. The model can be expressed with different range of coupling neighbors, without altering the symmetry of the system^44,50^. Based on the range of coupling, the model can be categorized as short-range, extended-range and long-range models.

### Geometry at finite temperature

The advances in the experimental realizations of the topological matter has made a significant effort in defining the topology at finite temperature. In this regard, there are efforts in defining the topological invariant through Uhlmann and interferometric phases (mixed states) as equivalent tool to Berry phase (pure state)^31^ in the finite temperature limit ($T\rightarrow 0$). Here we study the possibility of defining extended-range systems at finite temperature.

When a system is in contact with the thermal bath at temperature *T*, it can be defined by the Gibb’s state as^31^,

7 $$\begin{aligned} \rho (k)=\frac{e^{-H_k/T}}{Tr[e^{-H_k/T}]}=\frac{1}{2}\left( 1+\tanh \left( \frac{\Delta _k}{2T} \right) (\chi _i.\sigma _i) \right) , \end{aligned}$$

with $\Delta _k=2E(k,\textbf{M})$, which can be defined over the Bloch sphere using the polar coordinates $r=\sqrt{\chi _y^2+\chi _z^2}$ and $\phi =tan^{-1}\left( \frac{\chi _y(k)}{\chi _x(k)}\right)$. As the system is temperature dependent, the Bloch sphere is mapped to a sphere centered at the maximally mixed state. For the topological condition, this curve makes a closed loop and for non-topological the curve confines to a single side of the maximally mixed states respectively.

In case of Hermitian models, the method is straightforward, where the Gibbs’s state corresponds to a single parameter space $F(k,\textbf{M})$. In case of non-Hermitian models, the Gibb’s state can be expressed in terms of parameter spaces $F1(k,\textbf{M}),F2(k,\textbf{M})$ corresponding to individual exceptional points. Thus the effective geometry of the parameter space can be understood by the combined effect of $F1(k,\textbf{M})$ and $F2(k,\textbf{M})$.

**Uhlmann geometric phase** Here, the pure states in the Hilbert space $\mathscr {H}_A\bigotimes \mathscr {H}_B$ form a total space of fiber bundles over the mixed states of $\mathscr {H}_A$. The geometric phase can be achieved from parallelism condition over the base manifold such that every infinitesimal change $\delta k$ from the state $\psi (k)^{\dagger }$ to $\psi (k+\delta k)$ which equals the fidelity of density matrix^20–22^. i.e.,

8 $$\begin{aligned} |Tr(\psi (k)^{\dagger }\psi (k+\delta k))|^2=Tr\sqrt{\sqrt{\rho (k)}\rho (k+\delta k)\sqrt{\rho (k)}}, \end{aligned}$$

where $\rho (k)=\psi (k)\psi (k)^{\dagger }$ and has a gauge *U*(*N*) freedom such that $\rho \rightarrow \psi (k)U(N)U^{\dagger }(k)\psi ^{\dagger }(k)$ remains unchanged. With the limit $T\rightarrow 0$, the system drives towards pure state limit and the geometric phase becomes

9 $$\begin{aligned} \textrm{Tr}[\psi _{||}^{\dagger }(k)\psi _{||}(k)]= & {} \cos \left( \frac{1}{2}\oint dk\left( \partial _k\theta \right) \right) , \end{aligned}$$

with Uhlmann phase^31,60^

10 $$\begin{aligned} W_U= & {} Arg\left[ Tr[\psi _{||}^{\dagger }(k)\psi _{||}(k)]\right] =Arg\left[ \cos \left( \frac{1}{2}\oint dk\left( \partial _k\theta \right) \right) \right] . \end{aligned}$$

Thus the Uhlmann geometric phase in pure state limit is technically equivalent to the Berry phase (For detailed study, please refer “Method” section). This methodology can be generalized to both Hermitian and non-Hermitian systems.

For the Hermitian systems the method is straightforward where we have single closing point and the parameter space corresponding to $F(k,\textbf{M})$ gives the information of geometric phases. The method is slightly modified in the non-Hermitian systems, where we obtain two exceptional points. Thus, the Uhlmann phase can be calculated as the average of individual geometric phases corresponding to parameter spaces of $F1(k,\textbf{M})$ and $F2(k,\textbf{M})$, i.e., $W_U=\frac{W_{U1}+W_{U2}}{2}$.

### Hermitian Su-Schrieffer-Heeger chain

**Figure 1:**
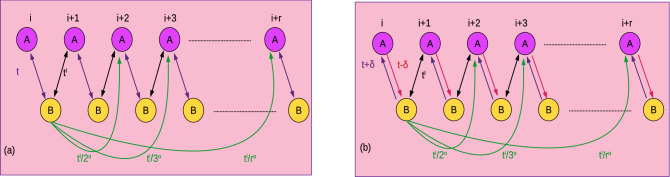
(Color online) Schematic representation of model Hamiltonian in the open boundary condition. (**a**) Hermitian SSH chain (**b**) non-Hermitian SSH chain. The extended-range coupling is introduced through intercell hopping, which decays as power law. The non-Hermiticity is introduced through the imbalance in the intracell hopping.

Here we consider 1D Su-Schrieffer-Heeger chain with extended-range of intercell hopping, i.e.,

11 $$\begin{aligned} H= & {} \sum _{j=1}^{L-l} t(c^{\dagger }_{j,a}c_{j,b}+c^{\dagger }_{j,b}c_{j,a}) +\sum _{j=1}^{L-l} \sum _{l=1}^{r} \frac{t^{\prime }}{l^{\alpha }}(c^{\dagger }_{j+l,a}c_{j,b}+c^{\dagger }_{j,b}c_{j+l,a}), \end{aligned}$$

where *t* is the intracell hopping and $t^{\prime }$ is the intercell hopping with power law decay. The term *l* represents the site index with $\alpha$ as the decay parameter. With the limit $l\rightarrow \infty$ (infinite neighbors) the model becomes long-range and as $\alpha \rightarrow \infty$ the model reduces to short-range (where the system contains only $W=1$ and $W=0$ phases). This model resembles the physics of another famous model Kitaev chain in the isotropic limit, where the hopping parameter (J) and pairing parameter ($\Delta$) decays with same manner $\frac{J}{l^{\alpha }}=\frac{\Delta }{l^{\alpha }}$^42,44^. The schematic representation is given in Fig. 1a. After Fourier transformation, we write BdG Hamiltonian in the pseudo-spin basis as in Eq. (1) with coefficients

12 $$\begin{aligned} \chi _x(k)= & {} t +t^{\prime }\sum _{l=1}^{r}\frac{\cos (kl)}{l^{\alpha }}, \chi _y(k)=t^{\prime }\sum _{l=1}^{r}\frac{\sin (kl)}{l^{\alpha }},\chi _z(k)=0. \end{aligned}$$

The model takes different phase diagrams and critical conditions based on the number of neighbors as shown in Fig. 4a–d. In this case, the quasi-energy dispersion and curvature function are real quantities given by,

13 $$\begin{aligned} E(k,\textbf{M})=\sqrt{(\chi _x)^2+(\chi _y)^2},F(k,\textbf{M})=\frac{\chi _y(k)\partial _k\chi _x(k)-\chi _x(k)\partial _k\chi _y(k)}{(\chi _x(k))^2+(\chi _y(k))^2}. \end{aligned}$$

At criticality, the energy dispersion vanishes and curvature function becomes non-analytic.

### Non-Hermitian Su-Schrieffer-Heeger chain

Here we construct non-Hermitian SSH chain by introducing an imbalance through the intracell hopping parameter. The Hamiltonian can be written as^75,77^,

14 $$\begin{aligned} H= & {} \sum _{j=1}^{L-l} (t-\delta )c^{\dagger }_{j,a}c_{j,b}+(t+\delta )c^{\dagger }_{j,b}c_{j,a} +\sum _{j=1}^{L-l} \sum _{l=1}^{r} \frac{t^{\prime }}{l^{\alpha }}(c^{\dagger }_{j+l,a}c_{j,b}+c^{\dagger }_{j,b}c_{j+l,a}), \end{aligned}$$

where $\delta$ is the imbalance term. With the limit $l\rightarrow \infty$ (infinite neighbors) the model becomes long-range and as $\alpha \rightarrow \infty$ the model reduces to short-range (where the system contains only $W=1$ and $W=0$ phases). The term $\frac{t^{\prime }}{l^{\alpha }}$ represents extended-range intercell coupling with $\alpha$ as the decay parameter as schematically represented in Fig. 1b. After Fourier transformation, the Hamiltonian can be written in spin basis as in Eq. (1), with coefficients,

15 $$\begin{aligned} \chi _x(k)= & {} t +t^{\prime }\sum _{l=1}^{r}\frac{\cos (kl)}{l^{\alpha }}, \chi _y(k)=t^{\prime }\sum _{l=1}^{r}\frac{\sin (kl)}{l^{\alpha }}+i\delta ,\chi _z(k)=0. \end{aligned}$$

respectively. Due to imbalance in the intracell hopping, the energy dispersion becomes complex and thus the non-Hermiticity is introduced into the model. For the current model, energy dispersion is given by

16 $$\begin{aligned} E(k,\textbf{M})= & {} \sqrt{(\chi _x)^2+(\chi _y^{re}(k)+\chi _y^{im}(k))^2}. \end{aligned}$$

The curvature function around exceptional points (0,$\pm \chi _y^{im}(k$)) are given by

17 $$\begin{aligned} F1(k,\textbf{M})= & {} \frac{(\chi _x^{re}(k)+\chi _y^{im}(k))\partial _k\chi _y^{re}(k)-\chi _y^{re}(k)\partial _k(\chi _x^{re}(k)+\chi _y^{im}(k))}{(\chi _x^{re}(k))^2+(\chi _y^{re}(k)+\chi _y^{im}(k))^2},\nonumber \\ F2(k,\textbf{M})= & {} \frac{(\chi _x^{re}(k)-\chi _y^{im}(k))\partial _k\chi _y^{re}(k)-\chi _y^{re}(k)\partial _k(\chi _x^{re}(k)+\chi _y^{im}(k))}{(\chi _x^{re}(k))^2+(\chi _y^{re}(k)-\chi _y^{im}(k))^2}, \end{aligned}$$

whose integral over the closed interval gives the WN. For the non-Hermitian case, WN is calculated as the average of individual WNs around the exceptional points^75^, i.e., $W=\frac{W_1+W_2}{2}$. The model exhibits a interesting property called non-Hermitian skin effect, due to which the phase diagram, winding number and localized modes show the sensitivity towards the choice of boundary conditions. Initially we present the geometric properties in the periodic boundary conditions and skin effect in the later sections.

## Results

In the section we study the mixed state behavior of topological models with different range of couplings. Here we take the study of pseudo-spin vectors and geometric phase for this purpose. The pseudo-spin (winding) vectors play an important role in defining the topology of a two level system. The number of time WVs rotate or wrap the center of parameter space gives the WN^42,81^. Here we consider the normalized WVs, which wrap the center of the maximally mixed states. For topological condition, WVs wrap equal to that of WN and for non topological they keeps bouncing at one side of the parameter space. For the phase transition condition, the WVs show a discontinuity in their flow (3D representation) or curvature line touches the center of the parameter space (2D representation). It is interesting that, at criticality, the angle $\tan \theta =\frac{\chi _y}{\chi _x}$ becomes indeterminate (0/0) as both $\chi _x,\chi _y\rightarrow 0$ resulting in an ill-defined topological invariant. This situation is similar in case of non-Hermitian systems, where the azimuthal angle is complex. The modified angles around the exceptional points can be written as $\tan (\theta _{1,2})=\frac{\chi _x^{(im)}+\chi _y^{(re)}}{\chi _x^{(re)}-\chi _y^{(im)}}$, which becomes indeterminate only if the terms $\chi _y^{(re)},\chi _x^{(re)}\pm \chi _y^{(im)}\rightarrow 0$.

The situation is little different in long-range interaction, where the WVs take the form of polylogarithmic functions. Here $\tan \theta = \frac{\chi _y}{\chi _x}$ becomes 0/0 at $k=\pi (\forall \alpha )$ and $k=0(\alpha >1)$ respectively. This is because, at $k=0$, the function $Li_{\alpha }(1)\propto \Gamma (\alpha -1)$ and remain divergent. Hence, for $\alpha <1$ the WVs show a discontinuity around $k=0$, resulting in the formation of removable singularities, whose integration gives the fractional WN.

Here we consider three different ranges to understand the interplay of neighboring coupling and mixed state behavior in topological (Hermitian/non-Hermitian) systems.

### Short-range couplings

If the coupling parameter has a strength only up to first neighbor, then the model can be called as short-range model, i.e., the decay parameter $\alpha \rightarrow \infty$. Here, we study the mixed state behavior of Hermitian and non-Hermitian SSH chain with short-range coupling.

#### Hermitian case

In this case, the Hamiltonian is given by Eq. (11) with $\alpha \rightarrow \infty$, whose Fourier transform yield Eq. (12) in the form

18 $$\begin{aligned} \chi _x(k)= & {} t +t^{\prime }\cos (k), \chi _y(k)=t^{\prime }\sin (k),\chi _z(k)=0. \end{aligned}$$

**Figure 2:**
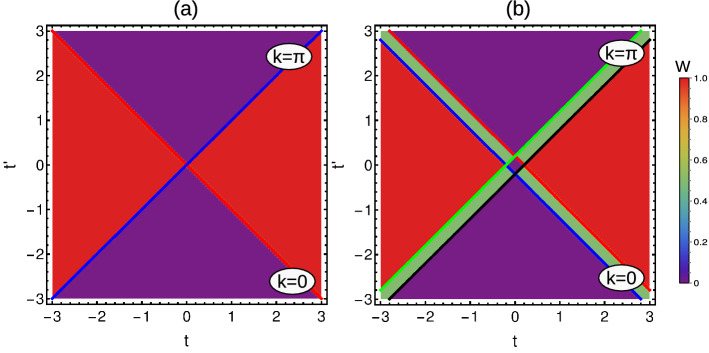
(Color online) Phase diagram of short-range SSH chain with periodic boundary condition (**a**) Hermitian case (**b**) Non-Hermitian case. In Hermitian case, we obtain only integer topological phases, whereas in non-Hermitian case, we obtain fractional phases along with integer topological phases.

The criticality occurs at $t=t^{\prime }(k=0)$ and $t=-t^{\prime }(k=\pi )$ by separating $W=0$ and $W=1$ phases as shown in Fig. 2a. The corresponding Gibb’s states are given by

19 $$\begin{aligned} \rho _x(k)= & {} \frac{1}{2}\left( 1+\tanh \left( \frac{2\sqrt{(t +t^{\prime }\cos (k))^2+(t^{\prime }\sin (k))^2}}{2T} \right) (t +t^{\prime }\cos (k)) \right) ,\nonumber \\ \rho _y(k)= & {} \frac{1}{2}\left( 1+\tanh \left( \frac{2\sqrt{(t +t^{\prime }\cos (k))^2+(t^{\prime }\sin (k))^2}}{2T} \right) (t^{\prime }\sin (k)) \right) , \end{aligned}$$

**Figure 3:**
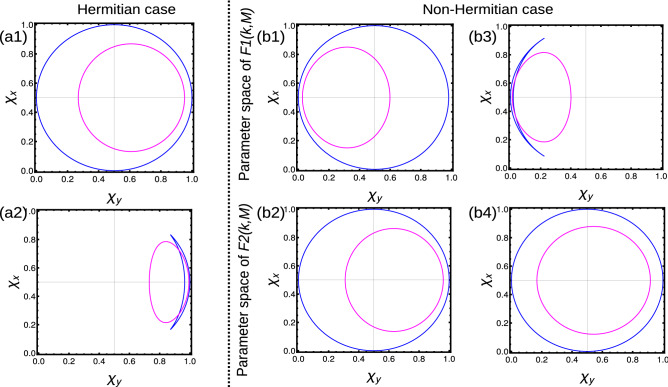
(Color online) Winding vector analysis of Hermitian and non-Hermitian short-range SSH chain with one neighbor coupling. The blue curve and magenta curves represent the zero temperature and $T=0.1$ calculated using Eqs. (18) and (19) (Hermitian) and Eqs. (20) and (22) (non-Hermitian) respectively. (**a1**, **a2**) Hermitian case with $W=1$ and $W=0$ case. (**b1**–**b4**) Non-Hermitian condition with $W=1$ and $W=1/2$ case.

At $T=0$, the WVs wrap the origin of the parameter space only one time for $W=1$ phases (Fig. 3a1), where as for $W=0$, the WVs do not wrap the origin (Fig. 3a2). With the introduction of the arbitrary temperature ($T=0.1$), the WVs show some modifications but the nature remains same as previous. The further increase in temperature, the curves gradually move towards one corner of the parameter space which may finally result in breakdown of topological properties. On the other hand, the Uhlmann phase become equal to Berry phase in the pure state limit $(T\rightarrow 0)$ (Table 1). Our results are consistent with the previous work Ref.^31^.

**Table 1:** A comparison of different pure and mixed state geometric phases of SSH chain for short-range coupling ($\alpha \rightarrow \infty$). The top and bottom tables represents the Hermitian and non-Hermitian SSH cases respectively. The Berry phase and Uhlmann phases are calculated through Eqs. (4) and (10) respectively. Here the geometric phases are quantized in the units of $\pi$ with $t^{\prime }=1$.

Region	Berry phase	Uhlmann phase
$t<-t^{\prime }, t>t^{\prime }$	0	0
$-t^{\prime }<t< t^{\prime }$	1	1
$t<-t^{\prime }-\delta, t>t^{\prime }+\delta$	0	0
$-t^{\prime }-\delta <t<-t^{\prime }+\delta, t^{\prime }-\delta<t< t^{\prime }+\delta$	1/2	1/2
$-t^{\prime }+\delta <t<t^{\prime }-\delta$	1	1

#### Non-Hermitian case

In this case, the Hamiltonian is given by Eq. (14) with the limit $\alpha \rightarrow \infty$, whose Fourier transform gives Eq. (15) in the form

20 $$\begin{aligned} \chi _x(k)= & {} t +t^{\prime }\cos (k), \chi _y(k)=t^{\prime }\sin (k)+i\delta ,\chi _z(k)=0. \end{aligned}$$

The criticality condition is given by $t=-t^{\prime }\pm \delta (k=0)$ and $t=t^{\prime }\pm \delta (k=\pi )$ which separate phases $W=0,1/2$ and $W=1$ as shown in Fig. 2b. Instead of single gap closing point, we get two exceptional points which also produce two parameter spaces for the WVs. The combined effect in the parameter spaces decides the topological behavior of the system. The EWVs are given by,

21 $$\begin{aligned} \chi _{Ex}(k)= & {} t +t^{\prime }\cos (k)\pm \delta , \chi _{Ey}(k)=t^{\prime }\sin (k),\chi _{Ez}(k)=0. \end{aligned}$$

and corresponding Gibb’s states are given by

22 $$\begin{aligned} \rho _{Ex}(k)= & {} \frac{1}{2}\left( 1+\tanh \left( \frac{2\sqrt{(t +t^{\prime }\cos (k)\pm \delta )^2+(t^{\prime }\sin (k))^2}}{2T} \right) (t +t^{\prime }\cos (k)\pm \delta ) \right) ,\nonumber \\ \rho _{Ey}(k)= & {} \frac{1}{2}\left( 1+\tanh \left( \frac{2\sqrt{(t +t^{\prime }\cos (k)\pm \delta )^2+(t^{\prime }\sin (k))^2}}{2T} \right) (t^{\prime }\sin (k)) \right) , \end{aligned}$$

At $T=0$, the EWVs encircle the origin once in both parameter space for $W=1$ case (Fig. 3b1,b2). For $W=1/2$, the EWVs encircle the origin of only one parameter space as shown in Fig. 3b3,b4. With the introduction of arbitrary temperature, the curve starts shifting towards one end of the parameter space. The higher temperature may destroy the topological properties of the system. The Uhlmann phase is given by Eq. (10), which becomes equal to Berry phase in the pure state limit $T\rightarrow 0$ (Table 1).

### Extended-range couplings

**Figure 4:**
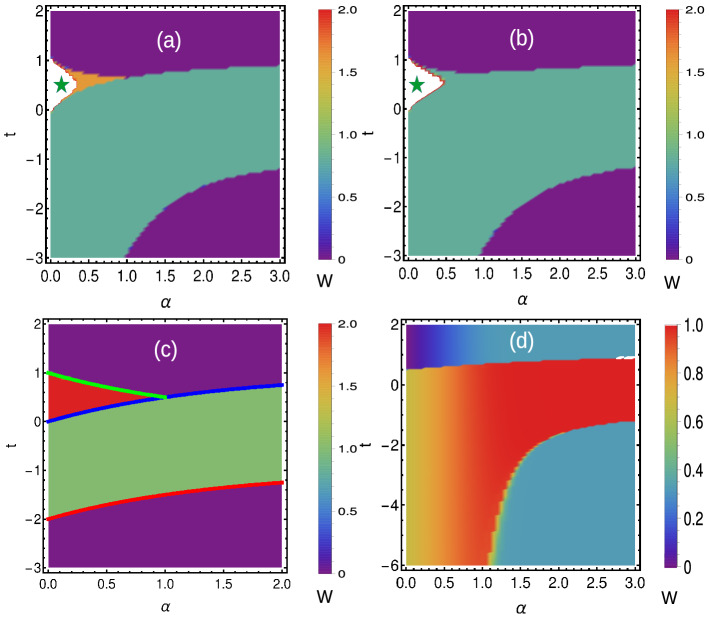
(Color online) Phase diagrams of Hermitian SSH chain with periodic boundary conditions. (**a**) Extended-range model with even number of neighbors. (**b**) Extended-range model with odd number of neighbors. (**c**) Simple extended-range model with two neighbors (**d**) Long-range model with infinite neighbors. Here the star symbol represents the region of higher WNs, which are less stable compared to their lower WNs.

When the coupling strength is more than one nearest neighbor, the model is called extended model^44^ and the phase diagram varies based on the number of coupling neighbors as shown in Figs. 4a–d and 6a–d. With the increase of neighbors, the possible WN also increases up to certain level and the model reduces to short-range limit with increasing value of decay parameter. This creates a staircase of topological transitions and we can observe a pattern of transitions among even-even and odd-odd WNs, based on the number of interacting neighbors^42^.

To understand the mixed state behavior, we consider the simple case $r=2$, where there are only two neighbor couplings for Hermitian and non-Hermitian situations.

#### Hermitian case

In this case, model Hamiltonian is given by Eq. (11) with $r=2$, whose Fourier transform is gives Eq. (12) in the form

23 $$\begin{aligned} \chi _x(k)= & {} t+t^{\prime }\left( \cos (k)+\frac{\cos (2k)}{2^{\alpha }}\right) , \chi _y(k)=t^{\prime }\left( \sin (k)+\frac{\sin (2k)}{2^{\alpha }}\right) , \chi _z(k)=0. \end{aligned}$$

The criticality condition is given by

24 $$\begin{aligned} k=0\rightarrow t=-t^{\prime }\left( 1+\frac{1}{2^{\alpha }}\right) , k=\pi \rightarrow t=-t^{\prime }\left( -1+\frac{1}{2^{\alpha }}\right) , k=\cos ^{-1}(-2^{\alpha -1})\rightarrow t=\frac{t^{\prime }}{2^{\alpha }}. \end{aligned}$$

which separate $W=0,1$ and $W=2$ phases. The phase diagram is given by Fig. 4c. The Gibb’s states corresponding to above parameter space are given by,

25 $$\begin{aligned} & \rho _{x} (k) = \frac{1}{2}\left( {1 + \tanh \left( {\frac{{2\sqrt {\left( {t + t^{\prime } \left( {\cos (k) + \frac{{\cos (2k)}}{{2^{\alpha } }}} \right)} \right)^{2} + \left( {t^{\prime } \left( {\sin (k) + \frac{{\sin (2k)}}{{2^{\alpha } }}} \right)} \right)^{2} } }}{{2T}}} \right)\left( {t + t^{\prime } \left( {\cos (k) + \frac{{\cos (2k)}}{{2^{\alpha } }}} \right)} \right)} \right), \\ & \rho _{y} (k) = \frac{1}{2}\left( {1 + \tanh \left( {\frac{{2\sqrt {\left( {t + t^{\prime } \left( {\cos (k) + \frac{{\cos (2k)}}{{2^{\alpha } }}} \right)} \right)^{2} + \left( {t^{\prime } \left( {\sin (k) + \frac{{\sin (2k)}}{{2^{\alpha } }}} \right)} \right)^{2} } }}{{2T}}} \right)\left( {t^{\prime } \left( {\sin (k) + \frac{{\sin (2k)}}{{2^{\alpha } }}} \right)} \right)} \right), \\ \end{aligned}$$

**Figure 5:**
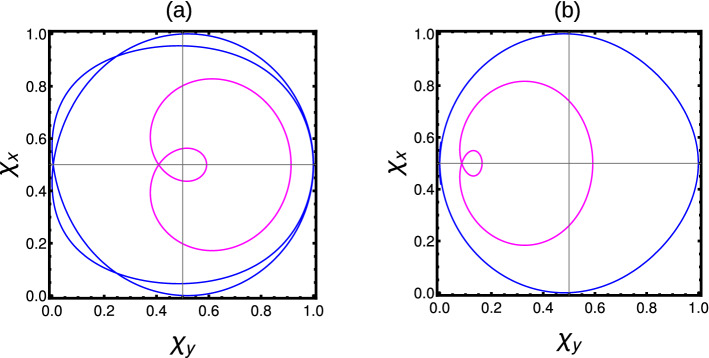
(Color online) Winding vector analysis of Hermitian extended-range SSH chain with two neighbors. The blue and magenta curves represent the zero temperature and $T=0.1$ calculated using Eqs. (24) and (25) respectively. (**a**) $W=2$ case (**b**) $W=1$ case.

At $T=0$, the WVs encircle the origin two (one) times for $W=2 (W=1)$ phases as shown in Fig. 5a,b. With the introduction of an arbitrary temperature, the enclosed area shrinks but the nature remains same. The further increase of the temperature results in the localization of curves towards a corner of the parameter space which results in the breakdown of the topological properties. The Uhlmann phase is given by Eq. (10), which shows a different behavior for extended-range coupling. The Uhlmann phase recognizes $W=0$ and $W=1$, but fails to recognize $W=2$. This result is also holds same to other extended-range models, with finite number of neighbor couplings. This is because, the Uhlmann phase fails to recognize multiple winding of curves around the center of parameter space. The results are presented in Table 2.

**Table 2:** A comparison of different pure and mixed state geometric phases of extended-range SSH chain for two nearest neighbors ($r=2$). The top and bottom tables represent the Hermitian and non-Hermitian SSH chains respectively. The Berry phase and Uhlmann phases are calculated through Eqs. (4) and (10) respectively. Here the geometric phases are quantized in the units of $\pi$ with $t^{\prime }=1$.

Region	Berry phase	Uhlmann phase
$$t<-t^{\prime }(1+\frac{1}{2^{\alpha }})$$$$t>-t^{\prime }(-1+\frac{1}{2^{\alpha }})$$	0	0
$$-t^{\prime }(1+\frac{1}{2^{\alpha }})$$$$<t<$$$$-t^{\prime }(-1+\frac{1}{2^{\alpha }})$$	1	1
$$-t^{\prime }(-1+\frac{1}{2^{\alpha }})$$$$<t<$$$$\frac{t^{\prime }}{2^{\alpha -1}}$$	2	0

Region	Berry phase $\left( W_B=\frac{W1+W2}{2}\right)$	Uhlmann phase $\left( W_U=\frac{W1+W2}{2}\right)$
$t<-\delta -1-\frac{1}{2^{\alpha }}$	$\frac{0+0}{2}=0$	$\frac{0+0}{2}=0$
$-\delta -1-\frac{1}{2^{\alpha }}<t<\delta -1-\frac{1}{2^{\alpha }}$	$\frac{0+1}{2}=\frac{1}{2}$	$\frac{0+1}{2}=\frac{1}{2}$
$\delta -1-\frac{1}{2^{\alpha }}<t<-\delta +1-\frac{1}{2^{\alpha }}$	$\frac{1+1}{2}=1$	$\frac{1+1}{2}=1$
$-\delta +1-\frac{1}{2^{\alpha }}<t<\delta +1-\frac{1}{2^{\alpha }}$ (for $\alpha <1$) (for $\alpha >1$)	$\frac{1+2}{2}=\frac{3}{2}$ $\frac{1+0}{2}=\frac{1}{2}$	$\frac{1+0}{2}=\frac{1}{2}$ $\frac{1+0}{2}=\frac{1}{2}$
$-\delta +1-\frac{1}{2^{\alpha }}<t<-\delta +\frac{1}{2^{\alpha }}$ (for $\alpha <0.5$)	$\frac{2+2}{2}=2$	$\frac{0+0}{2}=0$
$-\delta +\frac{1}{2^{\alpha }}<t<\delta +\frac{1}{2^{\alpha }}$ (for $\alpha <1$)	$\frac{2+0}{2}=1$	$\frac{0+0}{2}=0$
$t>\delta +\frac{1}{2^{\alpha }}$	$\frac{0+0}{2}=0$	$\frac{0+0}{2}=0$

#### Non-Hermitian case

In this case, model Hamiltonian is given by Eq. (14) with $r=2$, whose Fourier transform is gives Eq. (15) in the form

26 $$\begin{aligned} \chi _x(k)= & {} t+t^{\prime }\left( \cos (k)+\frac{\cos (2k)}{2^{\alpha }}\right) , \chi _y(k)=t^{\prime }\left( \sin (k)+\frac{\sin (2k)}{2^{\alpha }}\right) -i\delta , \chi _z(k)=0. \end{aligned}$$

**Figure 6:**
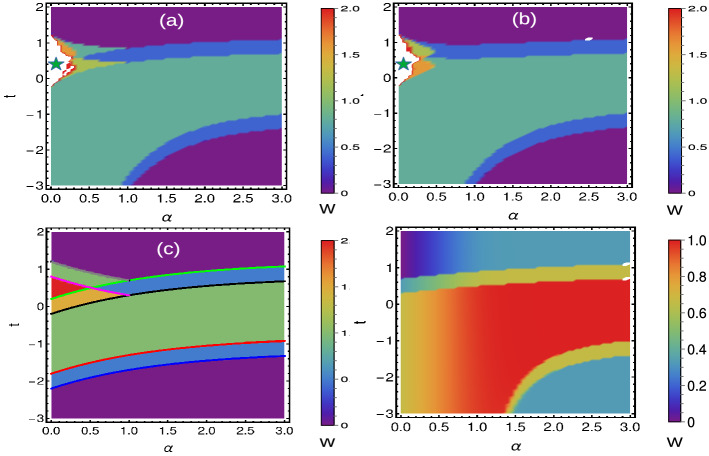
(Color online) Phase diagrams of non-Hermitian SSH chain with periodic boundary conditions. (**a**) Extended-range model with even neighbors. (**b**) Extended-range model with odd neighbors. (**c**) Simple extended-range model with two neighbors (**d**) Long-range model with infinite neighbors. Here the star symbol represents the region of higher WNs, which are less stable compared to their lower WNs.

The criticality occurs at

27 $$\begin{aligned} k= & {} 0\rightarrow t=-t^{\prime }\left( 1+\frac{1}{2^{\alpha }}\right) +\delta , t=-t^{\prime }\left( 1+\frac{1}{2^{\alpha }}\right) -\delta ,\nonumber \\ k= & {} \pi \rightarrow t=-t^{\prime }\left( -1+\frac{1}{2^{\alpha }}\right) +\delta , t=-t^{\prime }\left( -1+\frac{1}{2^{\alpha }}\right) -\delta ,\nonumber \\ k= & {} \cos ^{-1}(-2^{\alpha -1})\rightarrow t=\frac{t^{\prime }}{2^{\alpha }}+\delta ,t=\frac{t^{\prime }}{2^{\alpha }}-\delta . \end{aligned}$$

which separates $W=0,1/2,1,3,2,2,1$ and 0 topological phases as shown in Fig. 6c.

The EWVs are given by

28 $$\begin{aligned} \chi _{Ex}(k)= & {} t+t^{\prime }\left( \cos (k)+\frac{\cos (2k)}{2^{\alpha }}\right) \pm \delta , \chi _{Ey}(k)=t^{\prime }\left( \sin (k)+\frac{\sin (2k)}{2^{\alpha }}\right) , \chi _{Ez}(k)=0. \end{aligned}$$

The Gibb’s states corresponding to above parameter space are given by,

29 $$\begin{aligned} & \rho _{{Ex}} (k) = \frac{1}{2}\left( {1 + \tanh \left( {\frac{{2\sqrt {\left( {t + t^{\prime } \left( {\cos (k) + \frac{{\cos (2k)}}{{2^{\alpha } }}} \right) \pm \delta } \right)^{2} + \left( {t^{\prime } \left( {\sin (k) + \frac{{\sin (2k)}}{{2^{\alpha } }}} \right)} \right)^{2} } }}{{2T}}} \right)\left( {t + t^{\prime } \left( {\cos (k) + \frac{{\cos (2k)}}{{2^{\alpha } }}} \right) \pm \delta } \right)} \right), \\ & \rho _{{Ey}} (k) = \frac{1}{2}\left( {1 + \tanh \left( {\frac{{2\sqrt {\left( {t + t^{\prime } \left( {\cos (k) + \frac{{\cos (2k)}}{{2^{\alpha } }}} \right) \pm \delta } \right)^{2} + \left( {t^{\prime } \left( {\sin (k) + \frac{{\sin (2k)}}{{2^{\alpha } }}} \right)} \right)^{2} } }}{{2T}}} \right)\left( {t^{\prime } \left( {\sin (k) + \frac{{\sin (2k)}}{{2^{\alpha } }}} \right)} \right)} \right), \\ \end{aligned}$$

**Figure 7:**
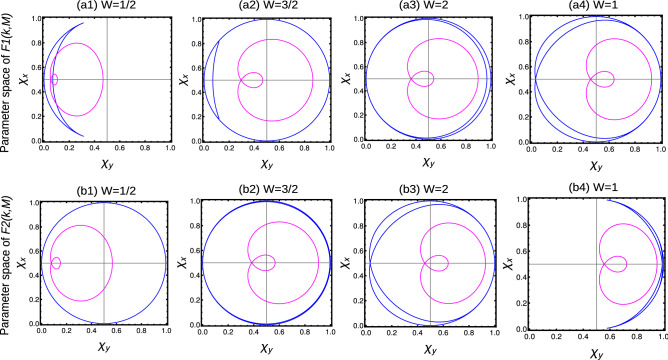
(Color online) Winding vector analysis of non-Hermitian extended-range SSH chain with two neighbors. The blue and magenta curves represent the zero temperature and $T=0.1$ calculated using Eqs. (28) and (29) respectively. (**a1**–**a4**) Behavior of extended winding vectors at parameter space corresponding to $F1(k,\textbf{M})$. (**b1**–**b4**) Behavior of extended winding vectors at parameter space corresponding to $F2(k,\textbf{M})$. The combined effect of $F1(k,\textbf{M})$ and $F2(k,\textbf{M})$ results in determining the topological property of the system.

In non-Hermitian extended models, the topological properties are determined by the average behavior of parameter space corresponding to $F1(k,\textbf{M})$ and $F2(k,\textbf{M})$. If the EWVs encircle the origin equal (unequal) number of times, results in integer (fractional) WNs. Sometimes the EWVs encircle one of the origin even (odd) number times and other origin zero times, resulting in integer (fractional) WNs. For $W=2$ case, the EWVs encircle the center of each parameter space two times (Fig. 7a3,b3), while they encircle each centers only once for $W=1$. For $W=1/2$ case, the EWVs encircle one of the center once and do not encircle the other (Fig. 7a1,b1). For $W=3/2$ case, the EWVs encircle one of the origin twice and the other only once (Fig. 7a2,b2). In some *special cases*, EWVs encircle the origin of one parameter space twice and do not encircle the other, which also result in $W=1$ (Fig. 7a4,b4). In non-Hermitian cases, the Uhlmann phase is given by the average of geometric phase with respect to parameter spaces $F1(k,\textbf{M})$ and $F2(k,\textbf{M})$, given by $W_U=\frac{W_{U1+W_{U2}}}{2}$ in the pure state limit ($T\rightarrow 0$). The Uhlmann phase recognizes $W=0,1/2$ and $W=1$ phases, but fails to recognize $W=3/2$ and $W=2$. This is due to the limitation of the geometry, that Uhlmann, approach do not recognizes the multiple number of windings around the origin. For the same reason, Uhlmann phase fails to recognize the above mentioned *special cases*, even though the resulting phase is $W=1$. A detailed study is given in Table 2. These results also holds for the extended-range models with higher number of neighboring couplings.

### Long-range couplings

With infinite number of coupling neighbors,the model becomes long-range and the pseudo-spin vectors are expressed in terms of polylogarithmic function. Expansions of polylogarithmic functions gives^82^,

30 $$\begin{aligned} Li_{\alpha }[e^{ik}]=\Gamma [1-\alpha ](-ik)^{\alpha -1}+\sum _{n=0}^{\infty }\frac{\zeta [\alpha -n]}{n!}(ik)^n \end{aligned}$$

where $\Gamma$ function becomes ill-defined for the region $\alpha <1$ around $k=0$. This creates a removable singularity in the parameter space, where the winding vectors covers only a half rotation around the axis and bounce back to the original configuration.Thus, they fail to give a integer winding number but produces fractional winding numbers.

#### Hermitian case

In this case, Hamiltonian is given by Eq. (11) with $l\rightarrow \infty$, whose Fourier transform gives Eq. (12) in the form

31 $$\begin{aligned} \chi _x(k)= & {} t+t^{\prime }\left( \frac{Li_{\alpha }[e^{i k}]+Li_{\alpha }[e^{-i k}]}{2}\right) , \chi _y(k)=t^{\prime }\left( \frac{Li_{\alpha }[e^{i k}]-Li_{\alpha }[e^{-i k}]}{2i}\right) ,\chi _z=0. \end{aligned}$$

where the term $Li_{\alpha }$ gives the polylogarithmic function^82^, which is the consequence of long-range effect. Here the criticality occurs at

32 $$\begin{aligned} k=0\rightarrow t= -t^{\prime }\text {Li}_{\alpha }(1), k=\pi \rightarrow t= -t^{\prime }\text {Li}_{\alpha }(-1). \end{aligned}$$

as shown in Fig. 4d. The Gibb’s states corresponding to above parameter space are given by,

33 $$\begin{aligned} & \rho _{x} (k) = \frac{1}{2}\left( {1 + \tanh \left( {\frac{{2\sqrt {\left( {t + t^{\prime } \left( {\frac{{Li_{\alpha } [e^{{ik}} ] + Li_{\alpha } [e^{{ - ik}} ]}}{2}} \right)} \right)^{2} + \left( {t^{\prime } \left( {\frac{{Li_{\alpha } [e^{{ik}} ] - Li_{\alpha } [e^{{ - ik}} ]}}{{2i}}} \right)} \right)^{2} } }}{{2T}}} \right)\left( {t + t^{\prime } \left( {\frac{{Li_{\alpha } [e^{{ik}} ] + Li_{\alpha } [e^{{ - ik}} ]}}{2}} \right)} \right)} \right), \\ & \rho _{y} (k) = \frac{1}{2}\left( {1 + \tanh \left( {\frac{{2\sqrt {\left( {t + t^{\prime } \left( {\frac{{Li_{\alpha } [e^{{ik}} ] + Li_{\alpha } [e^{{ - ik}} ]}}{2}} \right)} \right)^{2} + \left( {t^{\prime } \left( {\frac{{Li_{\alpha } [e^{{ik}} ] - Li_{\alpha } [e^{{ - ik}} ]}}{{2i}}} \right)} \right)^{2} } }}{{2T}}} \right)\left( {t^{\prime } \left( {\frac{{Li_{\alpha } [e^{{ik}} ] - Li_{\alpha } [e^{{ - ik}} ]}}{{2i}}} \right)} \right)} \right), \\ \end{aligned}$$

**Figure 8:**
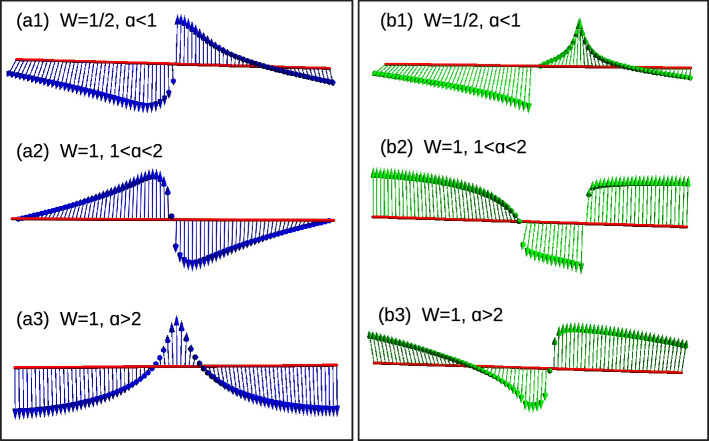
(Color online) Winding vectors representing the topological phases. (**a1**–**a3**) Zero temperature using Eq. (31) (**b1**–**b3**) Very small arbitrary temperature $T=0.1$ using Eq. (33) respectively.

Due to long-range effect, the WVs behave as $\sin (k)$ instead of $\sin (nk)$. Thus we obtain only two topological regions with $W=0$ and $W=1$. For $W=1$ the WVs encircle the axis once and for $W=0$ they do not encircle the axis. Due to polylogarithmic nature, $Li_{\alpha }(1)$ show a discontinuous region for $\alpha <1$, which results in an ill-defined topological region for $\alpha <1$^42,44,50^. Here we observe a discontinuity at $k=0$, which acts as a removable singularity and yields fractional WN ($W=1/2$) (Fig. 8a1). For $1<\alpha <2$, we observe a less populated WVs around $k=0$, which do not alters the resulting geometric phase (Fig. 8a2). For the range $\alpha >2$, we observe a homogeneous distribution of WVs, which is equivalent to the short-range limit, i.e., $W=1$ (Fig. 8a3). With the introduction of an arbitrary temperature, we observe a variation in density of WVs as shown in Fig. 8b1–b3. In the pure state limit ($T\rightarrow 0$), Uhlmann phase fails to recognize the fractional WN but it recognizes the integer WN as shown in Table 3.

**Table 3:** A comparison of different pure and mixed state geometric phases of long-range SSH chain for infinite neighbors ($r\rightarrow \infty$). The Berry phase and Uhlmann phase are calculated through Eqs. (4) and (10) respectively. Here the geometric phases are quantized in the units of $\pi$ with $t^{\prime }=1$. The top and bottom tables represents the Hermitian and non-Hermitian SSH chains respectively.

Region	Berry phase	Uhlmann phase	
$\alpha <1$ $t>-t^{\prime }(2^{1-\alpha }-1)\zeta [\alpha ]$	$-\pi /2$	Undefined	
$\alpha <1$ $t<-t^{\prime }(2^{1-\alpha }-1)\zeta [\alpha ]$	$\pi /2$	Undefined	
$\alpha >1$ $-t^{\prime } Li_{\alpha }(1)<\mu$ $<-2\lambda (2^{1-\alpha }-1)\zeta [\alpha ]$	1	1	
$\alpha >1$ $t<-t^{\prime } Li_{\alpha }(1)$ $t>-t^{\prime }(2^{1-\alpha }-1)\zeta [\alpha ]$	0	0	

Region	$\alpha$	Berry phase $\left( W_B=\frac{W1+W2}{2}\right)$	Uhlmann phase $\left( W_U=\frac{W1+W2}{2}\right)$
$t<-\delta -\text {Li}_{\alpha }(-1)$	$\alpha <1$	$\frac{0+1}{2}=\frac{1}{2}$	Undefined
$\delta -\text {Li}_{\alpha }(-1)$ $<t<$ $-\delta -\text {Li}_{\alpha }(-1)$	$\alpha <1$	$\frac{0+1/2}{2}=\frac{1}{4}$	Undefined
$t>\delta -\text {Li}_{\alpha }(-1)$	$\alpha <1$	$\frac{-1/2+0}{2}=-\frac{1}{4}$	Undefined
$-\delta -\text {Li}_{\alpha }(1)$ $<t<$ $\delta -\text {Li}_{\alpha }(1)$	$\alpha >1$	$\frac{0+1}{2}=\frac{1}{2}$	$\frac{0+1}{2}=\frac{1}{2}$
$\delta -\text {Li}_{\alpha }(-1)$ $<t<$ $-\delta -\text {Li}_{\alpha }(-1)$	$\alpha >1$	$\frac{1+0}{2}=\frac{1}{2}$	$\frac{1+0}{2}=\frac{1}{2}$
$\delta -\text {Li}_{\alpha }(1)$ $<t<$ $-\delta -\text {Li}_{\alpha }(-1)$	$\alpha >1$	$\frac{1+1}{2}=1$	$\frac{1+1}{2}=1$

#### Non-Hermitian case

In this case, Hamiltonian is given by Eq. (14) with $l\rightarrow \infty$, whose Fourier transform gives Eq. (15) in the form

34 $$\begin{aligned} \chi _x(k)= & {} t+t^{\prime }\left( \frac{Li_{\alpha }[e^{i k}]+Li_{\alpha }[e^{-i k}]}{2}\right) , \chi _y(k)=t^{\prime }\left( \frac{Li_{\alpha }[e^{i k}]-Li_{\alpha }[e^{-i k}]}{2i}\right) -i\delta ,\chi _z=0. \end{aligned}$$

The criticality occurs at

35 $$\begin{aligned} k= & {} 0\rightarrow t=\delta -t^{\prime }\text {Li}_{\alpha }(1),t=-\delta -t^{\prime }\text {Li}_{\alpha }(1).\nonumber \\ k= & {} \pi \rightarrow t=\delta -t^{\prime }\text {Li}_{\alpha }(-1),t=-\delta -t^{\prime }\text {Li}_{\alpha }(-1). \end{aligned}$$

as shown in phase diagram Fig. 6d. The EWVs are given by

36 $$\begin{aligned} \chi _{Ex}(k)= & {} t+t^{\prime }\left( \frac{Li_{\alpha }[e^{i k}]+Li_{\alpha }[e^{-i k}]}{2}\right) \pm \delta , \chi _{Ey}(k)=t^{\prime }\left( \frac{Li_{\alpha }[e^{i k}]-Li_{\alpha }[e^{-i k}]}{2i}\right) , \chi _{Ez}(k)=0. \end{aligned}$$

The Gibb’s states corresponding to above parameter space are given by,

37 $$\begin{aligned} & \rho _{{Ex}} (k) = \frac{1}{2}\left( {1 + \tanh \left( {\frac{{2\sqrt {\left( {t + t^{\prime } \left( {\frac{{Li_{\alpha } [e^{{ik}} ] + Li_{\alpha } [e^{{ - ik}} ]}}{2}} \right) \pm \delta } \right)^{2} + \left( {t^{\prime } \left( {\frac{{Li_{\alpha } [e^{{ik}} ] - Li_{\alpha } [e^{{ - ik}} ]}}{{2i}}} \right)} \right)^{2} } }}{{2T}}} \right)\left( {t + t^{\prime } \left( {\frac{{Li_{\alpha } [e^{{ik}} ] + Li_{\alpha } [e^{{ - ik}} ]}}{2}} \right) \pm \delta } \right)} \right), \\ & \rho _{{Ey}} (k) = \frac{1}{2}\left( {1 + \tanh \left( {\frac{{2\sqrt {\left( {t + t^{\prime } \left( {\frac{{Li_{\alpha } [e^{{ik}} ] + Li_{\alpha } [e^{{ - ik}} ]}}{2}} \right) \pm \delta } \right)^{2} + \left( {t^{\prime } \left( {\frac{{Li_{\alpha } [e^{{ik}} ] - Li_{\alpha } [e^{{ - ik}} ]}}{{2i}}} \right)} \right)^{2} } }}{{2T}}} \right)\left( {t^{\prime } \left( {\frac{{Li_{\alpha } [e^{{ik}} ] - Li_{\alpha } [e^{{ - ik}} ]}}{{2i}}} \right)} \right)} \right), \\ \end{aligned}$$

**Figure 9:**
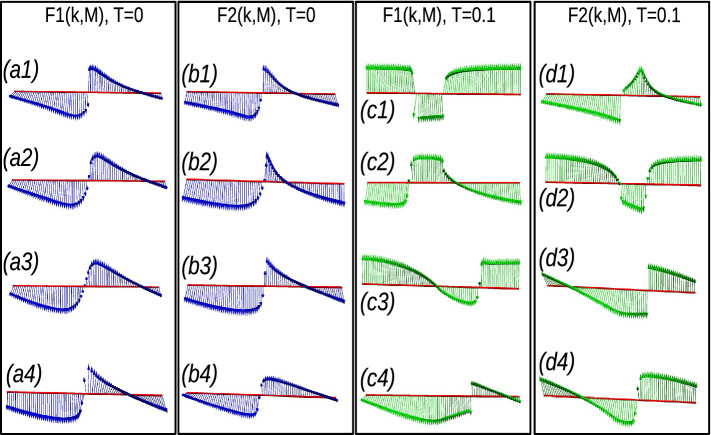
(Color online) Winding vectors representing the topological phases at Zero temperature (using Eq. (36)) and at a small arbitrary temperature $T=0.1$ (using Eq. (37)) respectively.

Here we can observe two kinds of fractional WNs. For $\alpha <1$ we find fractional WN as a result of polylogarithmic nature, (which are topologically ill-defined) and for $\alpha >1$ as a result of non-Hermiticity. At $T=0$, the EWVs corresponding to the parameter space corresponding to $F1(k,\textbf{M})$ and $F2(k,\textbf{M})$ show a discontinuity for $\alpha <1$ region, resulting in an ill defined topological invariant (Fig. 9a1,b1). But this this acts as a removable singularity and yield $W=1/4$ for $\alpha <1$ region. For $1<\alpha <2$, the EWVs encircle the axis once with a less populated arrows around $k=0$ (Fig. 9a2,b2). However, this does not influences the geometric phase of the system, and we obtain $W=1$ for $\delta -\text {Li}_{\alpha }(1)<t<-\delta -\text {Li}_{\alpha }(-1)$ region. For $\alpha >2$, we get the short-range limit with $W=1$ where the EWVs encircle the axis of both the parameter space (Fig. 9a3,b3). For the region ${\mp }\delta -\text {Li}_{\alpha }({\mp }1)<t<\pm \delta -\text {Li}_{\alpha }(\pm 1)$, we get $W=1/2$ where the EWVs encircle the axis of any one of the parameter space (Fig. 9 a4,b4). With the introduction of arbitrary temperature, the EWVs show the modifications as shown in Fig. 9 (Fig. 9c1–c4,d1–d4). In the pure state limit $T\rightarrow 0$, the Uhlmann phase recognizes the integer and fractional phases for $\alpha >1$ but fails to recognize the phases in the region $\alpha <1$. A detailed study is given in Table 3.

From the above study, we understand the limitation of Uhlmann phase in explaining the topological invariants at finite temperature, especially for extended-range models. In extended-range (Hermitian and non-Hermitian) models, the Uhlmann phase fails to reproduce the phase diagram at pure state limit. Here we use another important approach, interferometric geometric phase to explain the finite temperature behavior of extended-range topological models.

### Interferometric geometric phase

Here a different geometric phase for mixed states is introduced through the concept of interferometer^83^. The purification of normalized of state $|\omega \rangle \in \mathscr {H}_{\omega }$ is

38 $$\begin{aligned} |\omega \rangle =\sum _{i}\sqrt{p_i}|\psi _i\rangle \otimes |\partial _k\psi _i{\dagger }\rangle , \end{aligned}$$

with $\mathscr {H}_{\omega }=\mathscr {H}_S \otimes \mathscr {H}_A$ and $|\psi _i\rangle \in \mathscr {H}_A$. Here $\mathscr {H}_S$ is the Hilbert space of the system and $\mathscr {H}_A$ is the Hilbert space spanned by the ancillary states with *i* dimensions. By tracing over the ancillary states, we write the density matrix as, $\rho =Tr_A(|\omega \rangle \langle \omega |)$. By parameterizing the density operators with a continuous parameter *k*, such that the eigenvalues of $\rho (k)$ for each *k* yield non-degenerate values,

39 $$\begin{aligned} \rho (k)=\sum _{i}p_i(k)|\psi _i\rangle \langle \psi _i(k)|. \end{aligned}$$

The eigenstates evolve in parallel manner such that two infinitesimally separated eigenstates in $\mathscr {H}_s$ show parallel transport in order to fix the phase ambiguity of $|\omega \rangle$ through gauge fixing. The parallelism is given by

40 $$\begin{aligned} \langle \psi _i(k)|\partial _k\psi _i(k)\rangle =0, \end{aligned}$$

which yield the interferometric phase of $\rho (k)$ across the Brillouin zone. i.e.,

41 $$\begin{aligned} \theta _g=Arg[\sum _{i}\sqrt{p_i(0)p_i(2\pi )}\langle \psi _i(0)|V_i(2\pi )|\psi _i(2\pi )\rangle ], \end{aligned}$$

where $V_i(2\pi )=exp[-\oint dk^{\prime }\langle \psi _i(k^{\prime })|\partial _{k^{\prime }}\psi _i(k^{\prime })\rangle ]$. It is to be noted that, the state $|\psi _k\rangle$ is affected by parallel transport, while the ancillary states $|\psi _{k^{\prime }}\rangle$ are not. Hence, the interferometric phase reduces to Berry phase only if $\rho (k)$ is a parameterized density operator of pure states^31,60^.

**Figure 10:**
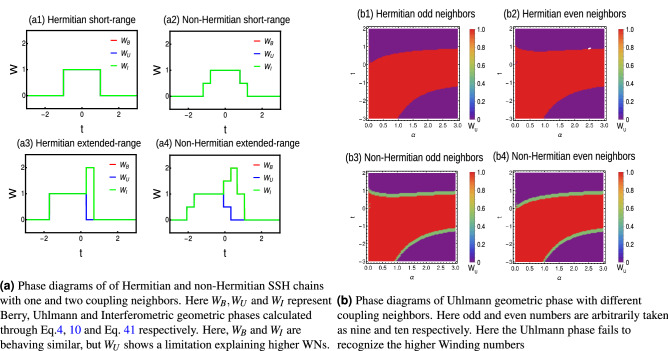
(Color online) (**a**) Comparison of different geometric phases. (**b**) Behavior of Uhlmann phase for higher coupling neighbors.

Thus, we can observe the higher WN as well as fractional WNs in the pure state limit. This geometric phase overcomes the limitations of Uhlmann phase and can be effectively used as topological measure in Hermitian and non-Hermitian systems. For the Hermitian system, the method remains straightforward. For the non-Hermitian systems, the interferometric phase can be calculated for the individual parameter spaces corresponding to $F_{1,2}(k,\textbf{M})$ and combined effect can be observed through the average $\frac{W_{I1}+W_{I2}}{2}$. Thus, we understand that interferometric geometric phase is a better tool to study the mixed state behavior of extended-range (Hermitian and non-Hermitian) topological models. A comparison is given in Fig. 10.

### Topology at gapless condition

Localization is an important property which has direct relation with the topological invariant. We can observe a one to correspondence between the number of localized modes and neighbor coupling up to some extent^44^. However, increase in the number of neighbor coupling result in the generation of higher winding numbers. With the increase of decay parameter, we observe a staircase of topological transitions among corresponding even-even and odd-odd winding numbers^42^. The bulk phases contain localized modes, protected by certain discrete symmetries. This naturally exhibits bulk-boundary correspondence and it is believed that, bulk gap is necessary to exhibit bulk-boundary correspondence. Recently, there have been observations of localization even at criticality exhibiting bulk-boundary correspondence, signaling the bulk-boundary correspondence even in the absence of bulk gap^72,73,84^. By definition, topological invariant is ill-defined at criticality, but there are efforts to define topology at criticality through different means. Here we consider a case of localization at criticality and define the topological invariant by excluding the infinitesimal neighborhood of singular points in the Brillouin zone^64^.

42 $$\begin{aligned} W=\left( \frac{1}{2\pi }\right) \lim _{\delta \rightarrow 0}\int _{\forall i:|k-k_i|>\delta }\frac{\partial \phi _{1,2}(k)}{\partial k}dk, \end{aligned}$$

where $\{k_i\}$ is the set of critical points in the momentum space. For the Hermitian case, we find a unique behavior at $k=\pi$ criticality. The critical line $\mu =-t^{\prime }(-1+\frac{1}{2^{\alpha }})$ line contains a multi-critical point at $\alpha =1$, where the critical line $\alpha <1$ witness the transition $W:2\rightarrow 1$ and $\alpha >1$ witnesses $W:0\rightarrow 1$ respectively (Fig. 4c). During the transition $W:2\rightarrow 1$, out of two edge modes, one localizes at criticality and the other transfers to the $W=1$ gapped phase. Thus, on the same critical line, we observe a localized mode for $\alpha <1$ and non localization for $\alpha >1$, which creates a topological transition along criticality across a multi-critical point^72,73,84^.

**Figure 11:**
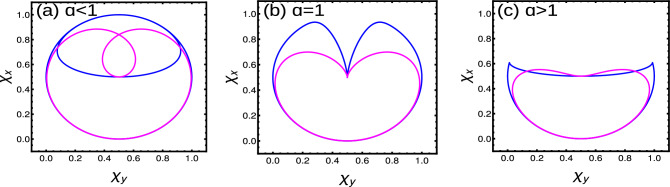
(Color online) Behavior of winding vectors of Hermitian SSH chain witnessing the topological transition along the critical line $t=-t^{\prime }(-1+\frac{1}{2^{\alpha }})$ for $k=\pi$. The blue curve represents the zero temperature and magenta represents $T=0.1$ respectively.

For the region $\alpha <1$, the WVs show two loops, with the inner one touching the center of the parameter space (Fig. 11 a), while for $\alpha >1$, WVs show a single loop touching the center (Fig. 11b). The change in configuration occurs at $\alpha =1$ as shown in Fig. 11c. Even though, all the configurations are occurring on the line $t=-t^{\prime }(-1+\frac{1}{2^{\alpha }})$, the topological behavior of them are different. Due to the localization property, we observe different WNs even at criticality. But at a finite temperature, Uhlmann phase does not recognizes the WNs at criticality. This limitation can be overcome by using interferometric phase, where we can recognize the WNs at criticality. The comparison of geometric phases are given in Table 4.

**Table 4:** A comparison of different pure and mixed state geometric phases of Hermitian SSH chain witnessing the topological transition along the critical line $t=-t^{\prime }(-1+\frac{1}{2^{\alpha }})$. Here the geometric phases are quantized in the units of $\pi$ with $t^{\prime }=1$. The Berry phase, Uhlmann and interferometric phases are calculated through Eqs. (4), (10) and (41) respectively.

Region	Berry phase	Uhlmann phase	Interferometric phase
$t<-t^{\prime }(-1+\frac{1}{2^{\alpha }})$ for ($\alpha <1$)	$\frac{3}{2}$	0	$\frac{3}{2}$
$t<-t^{\prime }(-1+\frac{1}{2^{\alpha }})$ for ($\alpha =1$)	1	0	1
$t<-t^{\prime }(-1+\frac{1}{2^{\alpha }})$ for ($\alpha >1$)	$\frac{1}{2}$	0	$\frac{1}{2}$

## Non-Hermitian skin effect

So far, we have constructed the phase diagram and calculated the topological invariant by assuming the periodic boundary condition to Hermitian and non-Hermitian SSH models. Under general conditions, topological invariant is calculated using Bloch band structure and is in good agreement with the number of localized edge modes. This scenario is true in Hermitian systems, while non-Hermitian systems behave differently. Non-Hermitian systems are sensitive to the boundary conditions, which produce different phase diagram for periodic and open boundary conditions. This behavior is due to the phenomenon called non-Hermitian skin effect, which signals the necessity of the construction of ‘non-Bloch topological invariant’. Due to non-Hermitian skin effect, the topologically protected edge modes depends on the spacial dimension and the symmetry class rather than the topological invariant^34^. The bulk-edge correspondence holds good for the open boundary condition and this creates a necessity to define topological invariant in open chain. In 2018, Yao et al, formulated this framework^77^ which works on the generalize Brillouin zone and topological invariant is given by

43 $$\begin{aligned} W_L=\oint \frac{dk}{4\pi i}Tr\left[ \sigma _zH^{-1}(k)\partial _kH(k)\right] . \end{aligned}$$

(The construction of generalized Brillouin zone is given in “Method” section). The current form of the modified winding number is due to the presence of chiral symmetry. Under the periodic boundary conditions, bulk Hamiltonian is given by Eq. (1) with $k\in \left[ 0,2\pi \right]$, whereas under open boundary condition Hamiltonian is given with $k\in \left[ 0,2\pi \right)$. The presence of sub lattice symmetry creates $\mathbb {Z}$ topological phase in the presence of line gap complex energy spectrum. The topological invariant has the similar form of that of Hermitian conditions. For the region with point gap complex energy spectrum, there exists $\mathbb {Z}\oplus \mathbb {Z}$ topological phases, and invariant is given by,

44 $$\begin{aligned} W_P=\oint _{BZ}\frac{dk}{2\pi i}\frac{d}{dk}\ln \text {Det}(H(k)). \end{aligned}$$

The $W_P$ becomes zero for an integer $W_L$, whereas $W_P$ becomes integer value for fractional $W_L$.

**Figure 12:**
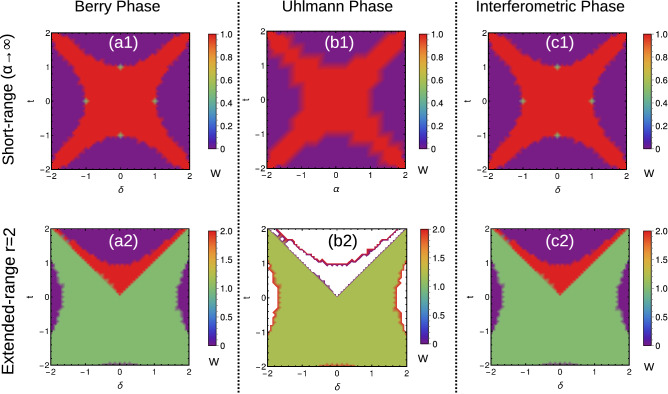
(Color online) Phase diagrams of non-Hermitian SSH chain in generalized Brillouin zone with $t^{\prime }=1$ and $\alpha =0.1$ (the construction of generalized Brillouin zone is given in “Method” section). The geometric phases of short-range ($\alpha \rightarrow \infty$) and extended-range ($r=2$) models are represented in pure and mixed state forms. The Uhlmann phase shows some limitation in defining the geometric phases at finite temperature.

Here we present the geometric phases of non-Hermitian SSH chain with different range of couplings (Fig. 12), which is different than its counterpart in periodic boundary condition (Fig. 12a1,a2). It clearly shows the sensitivity of phase diagram, towards the choice of boundary conditions. In generalized Brillouin zone, there can occur a significant calculation errors in defining the topological invariant for extended-range due to various reasons^89,90^. In addition, the Uhlmann phase shows a limitation in defining the geometric phase at finite temperature. The higher winding numbers are not defined by the Uhlmann phase and the phase boundary does not shows an exact point of transition (Fig. 12b1,b2). Instead, the phase is not exactly integer quantized (at least in extended-range) add the phase boundary is spreaded. On the other hand the interferometric phase shows the similar behavior that of Berry phase in the finite temperature ($T\rightarrow 0$) limit (Fig. 12c1,c2). However, so far the physical interpretation of Uhlmann phase has not been revealed in an extensive way, which creates a difficulty in interpreting the physical meaning of geometric phases at finite temperature. The Uhlmann phase also has a limitation in establishing the relation between the geometry at finite temperature and the bulk-edge correspondence^23,31^. The Uhlmann phase works on the principle of the parallel transport of density matrices at finite temperature, thus it recognized the critical temperature and the topological region, while it fails to determine the number of edge modes due to the memory effect^31,60^. On the other hand, the interferometric phase works straightforwardly based on the principle of ancillary states unlike Uhlmann phase (which works on operator method), and behaves similar to the Berry phase at $T\rightarrow 0$ limit. However, one can predict the criticality, critical temperature and geometric phases of mixed state topological systems through different means (both for periodic and open boundary conditions), but the understanding towards the bulk-edge correspondence needs some more study.

## Discussion

Mixed state behavior of quantum system is an efficient way to understand the thermal fluctuations, especially to define the topology at finite temperature ($T\rightarrow 0$). There are a few studies in literature to understand the finite temperature limit of topological system with short-range and long-range interactions. Here we made an attempt to understand the behavior of extended topological models in finite temperature limit. We have extended our observations to the non-Hermitian topological system to understand the geometry and geometric phases at finite temperature.

Introduction of extended range interaction in a topological system is an efficient way to generate the higher WNs, which is an static way. The similar effect can be done by the quenching/periodic driving, which is a dynamical method. In an equilibrium topological system, generally the WN is in correspondence with the localized edge modes in the gapped phases (at least up to some range $r<L/2$. Sometimes this may not be true if certain symmetries like time reversal is broken). Here we made an effort to study the extended topological chains in the finite temperature limit. We have used the mixed geometric phases like Uhlmann and interferometric phases to understand the topology at finite temperature. We have found that Uhlmann approach has a limitation to define the higher WNs and this can be verified by defining the winding vectors in the form of Gibb’s state. We use interferometric geometric phase to find the geometric phase at finite temperature as shown in Fig. 10. Long-range topological models are the platform to realize the massive edge modes (along with Majorana zero modes), which can also effectively used as topological qubit. Here we obtain fractional WNs for the region where massive edge modes dominate. We carry out finite temperature and observe that the Uhlmann phase has a limitation in defining the fractional WNs. The authors of Ref.^60^ have worked on similar model and mentioned a different undefined region for Uhlmann phase, due to the nature of superconducting pairing term.

Non-Hermiticity is a prominent area of quantum mechanics and here we have adopted biorthonormal basis vectors to define the topology of the models. The concept can be extended to the mixed states and hence it is possible to understand the geometric phases of non-Hermitian models at finite temperature. However, the non-Hermitian topological models are quite different than the Hermitian systems and have a separate set of periodic table of symmetry protected classes. Moreover, they are sensitive to the boundary condition, and phase diagram can differ according to that. Here we have analyzed chiral non-Hermitian SSH chain and their topology at finite temperature. We observe that the Uhlmann phase recognizes the fractional and integer phases in the short-range limit, but fails to recognize the same in extended-range. It is also to be noted that the similar situation has been observed in the open boundary condition (in the presence of non-Hermitian skin effect), where the Uhlmann phase shows a limitation in defining the higher winding numbers.

As per the traditional definition of topology, WN can be well defined in the gapped phases and ill-defined at the gapless regions. But the experimental and theoretical study at criticality strengthens the possibility of localized states even at criticality. This creates a need of defining topology at criticality and we adopted modified definition to address this issue. The study of Uhlmann phase shows a limitation in defining topological invariant at criticality and fails to recognize the transition among gapless regions across a multi-critical point.

To conclude, we have analyzed the mixed state behavior of Hermitian and non-Hermitian topological models and tried to calculate the geometric phase at $T\rightarrow 0$ limit. Among the study of geometric phase for mixed states, Uhlmann phase has showed a limitation in defining topological invariant for extended-range at finite temperature. This limitation can be overcome by the interferometric phase in the pure state limit. We also extend our study to non-Hermitian models and analyze the limitation of Uhlmann phase in defining the topological invariants for extended-range models. We have analyzed the geometric phases at open boundary condition and tried to understand the behavior of geometric phases in the presence of non-Hermitian skin effect. We understand that the physics of Uhlmann phase is complicated and there needs further study in this regard. We agree with the statement of Viyuela et al.^29^, and Anderson et al.^31^ that the Uhlmann phase does not determine the fate of edge modes at finite temperature’ and we extend validation of this statement to the extended range interaction of Hermitian and non-Hermitian models. We also find that interferometric phase can serve as an efficient tool calculate the topology of (Hermitian and non-Hermitian) extended-range models.

## Method

### Derivation of effective winding number for non-Hermitian systems

The generalized expression for WN is given by the Eq. (4), with periodic boundary condition the expression is

45 $$\begin{aligned} W=\left( \frac{1}{2\pi }\right) \int _{-\pi }^{\pi }\frac{\chi _x\partial _k\chi _y-\chi _y\partial _k\chi _x}{\chi _x^2+\chi _y^2} dk. \end{aligned}$$

Due non-Hermiticity, the Eigen vectors of the Hamiltonian can be written as^75^

46 $$\begin{aligned} W=\left( \frac{1}{2\pi }\right) \int _{-\pi }^{\pi }\frac{\langle \varphi (k)|i\partial _k|\psi (k)\rangle }{\langle \varphi (k)|\psi (k)\rangle }dk, \end{aligned}$$

where

47 $$\begin{aligned} \langle \varphi |=\frac{1}{\sqrt{2}} \left( \begin{array}{l} \frac{\chi _x+i\chi _y}{\sqrt{\chi _x^2+\chi _y^2}}\\ -1 \end{array} \right) ^T, |\psi \rangle =\frac{1}{\sqrt{2}} \left( \begin{array}{l} \frac{\chi _x-i\chi _y}{\sqrt{\chi _x^2+\chi _y^2}}\\ -1 \end{array}. \right) \end{aligned}$$

The non-Hermiticity induces the complexity in winding vectors(at in least one component) with the criticality condition $\chi _x^2+h\chi _y^2=0$. Here we observe exceptional points (at least two), instead of a single Dirac cone (band gap closing point), whose position can be understood by the complex analysis as,

48 $$\begin{aligned} \chi _x^{re}(k)=-\chi _y^{im}(k)&\text {and}&\chi _y^{re}(k)=\chi _x^{im}(k)\nonumber \\&\text {or}&\nonumber \\ \chi _x^{re}(k)=\chi _y^{im}(k)&\text {and}&\chi _y^{re}(k)=-\chi _x^{im}(k) \end{aligned}$$

**Figure 13:**
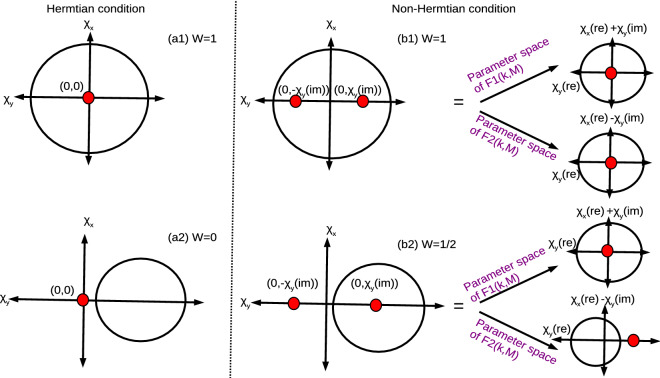
(Color online) Winding vector behavior of Hermitian and non-Hermitian systems. A extended winding vector can be constructed by using Eq. (6). A pseudo-spin parameter space corresponding to two exceptional points can be equivalently written as the sum of parameter spaces with modified winding vectors.

As our model contains imaginary component only in $\chi _y$ term, the exceptional points are located at $(0, \chi _y^{im})$ and $(0, -\chi _y^{im})$ under the criticality condition $\chi _x^2+\chi _y^2=0$. Due to the non-Hermiticity, each exceptional point induces its own origin of pseudo-spin space and corresponding winding vectors. Thus we work on extended winding vectors to understand the non-Hermitian effect in the parameter space. i.e.,

49 $$\begin{aligned} \chi _x(extended)= & {} \chi _x^{re}(k)\pm \chi _y^{im}(k),\nonumber \\ \chi _y(extended)= & {} \chi _y^{re}(k), \end{aligned}$$

which correspond to parameter space $F_1(k,\textbf{M})$ and $F_2(k,\textbf{M})$ respectively (Fig. 13).

As a result of non-Hermiticity the azimuthal angle becomes complex, i.e., $\phi =\phi ^{re}+i\phi ^{im}$, where the real and imaginary parts contribute to the argument and amplitude respectively. Thus the complex angle can be calculated as,

50 $$\begin{aligned} e^{i2\phi }= & {} \frac{1+i\tan (\phi )}{1-i\tan (\phi )}=\frac{\chi _x+i\chi _y}{\chi _x-i\chi _y}, e^{-i2\phi }=\left| \frac{\chi _x+i\chi _y}{\chi _x-i\chi _y} \right| , e^{-i2\phi _r}=\frac{\frac{\chi _x+i\chi _y}{\chi _x-i\chi _y} }{\left| \frac{\chi _x+i\chi _y}{\chi _x-i\chi _y} \right| },\nonumber \\ \tan (2\phi _r)= & {} \frac{Im(\frac{\chi _x+i\chi _y}{\chi _x-i\chi _y}) }{Re(\frac{\chi _x+i\chi _y}{\chi _x-i\chi _y} )} =\frac{\tan (\phi _1)+\tan (\phi _2)}{1-\tan (\phi _1)\tan (\phi _2)}=\tan (\phi _1+\phi _2) \end{aligned}$$

where $\tan (\phi _1)=\frac{\chi _y^{re}(k)+\chi _x^{im}(k)}{\chi _x^{re}(k)+\chi _y^{im}(k)}$ and $\tan (\phi _2)=\frac{\chi _y^{re}(k)+\chi _x^{im}(k)}{\chi _x^{re}(k)-\chi _y^{im}(k)}$. Thus azimuthal angle $\phi _1(\phi _2)$ corresponding to first (second) exceptional point can be expressed in real angles. The integration of curvature functions $F1(k,\textbf{M}),F2(k,\textbf{M})$ corresponding these azimuthal angles give the WN as i.e.,

51 $$\begin{aligned} W=\frac{1}{2}\left( \frac{1}{2\pi }\right) (\oint \partial _k\phi _1dk+\oint \partial _k\phi _2dk). \end{aligned}$$

Encircling of these extended winding vectors around the centers of both the parameter space (equal time) yields integer WN. If the encircling is only around one of them yields fractional and neither of them yields $W=0$ respectively.

### Generalized Brillouin zone and topological invariant

Due to the existence of non-Hermitian skin effect, the topological phase diagram becomes sensitive to boundary conditions. Here we adopt the non-Block band theory to treat the topological models under open boundary conditions^85^. For a short-range model (Eq. (14) with $\alpha \rightarrow \infty$), the real space Eigen equation leads

52 $$\begin{aligned} t^{\prime }\psi _{n-1,B}+(t+\delta )\psi _{n,B}= & {} E\psi _{n,A}\nonumber \\ t^{\prime }\psi _{n+1,A}+(t+\delta )\psi _{n,A}= & {} E\psi _{n,B} \end{aligned}$$

Considering the ansatz $|\psi \rangle =\sum _{j}|\phi ^{(j)}\rangle$, where each $|\phi ^{(j)}\rangle$ takes the form (omitting the *j* index temporarily) $(\phi _{n,A},\phi _{n,B})=\beta ^n(\phi _{A},\phi _{B})$. We obtain the relation

53 $$\begin{aligned} ((t+\delta )+t^{\prime }\beta ^{-1})\phi _{B}=E\phi _{A}\nonumber \\ ((t+\delta )+t^{\prime }\beta )\phi _{A}=E\phi _{B} \end{aligned}$$

the energy equation is given by

54 $$\begin{aligned} ((t+\delta )+t^{\prime }\beta ^{-1}) ((t+\delta )+t^{\prime }\beta )=E^2 \end{aligned}$$

Under the limit $E\rightarrow 0$, we obtain the roots

55 $$\begin{aligned} \beta _{1,2}^{E\rightarrow 0}=-\frac{t-\delta }{t^{\prime }},-\frac{t^{\prime }}{t+\delta } \end{aligned}$$

Restoring the *j* index, we obtain

56 $$\begin{aligned} \frac{((t+\delta )+t^{\prime }\beta ^{-1})}{E}\phi _{B}^{(j)}=\phi _{A}^{(j)}\nonumber \\ \frac{((t+\delta )+t^{\prime }\beta )}{E}\phi _{A}^{(j)}=\phi _{B}^{(j)} \end{aligned}$$

Thus the general solution for $\psi$ can be written as linear superposition of $\phi$ as $\psi =\sum _{n}\beta ^{(n)}\phi$. For the spectrum in thermodynamic limit, the bulk-Eigenstates follow the rule $|\beta _1|=|\beta _2|$, which leads to

57 $$\begin{aligned} |\beta _j|=r_0\equiv \sqrt{\left| \frac{t-\delta }{t+\delta }\right| } \end{aligned}$$

For the case $r=1$, the non-Hermitian model behaves similar to that of the Hermitian counterpart^88^.

*Topological invariant* Due to non-Bloch band structure, the components get modified as $e^{ik}\rightarrow \beta ,e^{-ik}\rightarrow \beta ^{-1}$ and the Hamiltonian is given by

58 $$\begin{aligned} H(\beta )=(t-\delta +t^{\prime }\beta ^{-1})\sigma _-+(t+\delta +t^{\prime }\beta )\sigma _+. \end{aligned}$$

where $\sigma _{\pm }=\frac{(\sigma _x+i\sigma _y)}{2}$. Here the *k* term takes the complex value $k\rightarrow k-i\ln (r_0)$. The energy relations are given by

59 $$\begin{aligned} H(\beta )|u_R\rangle =E(\beta )|u_R\rangle ,H^{\dagger }(\beta )|u_R\rangle =E^*(\beta )|u_R\rangle , \end{aligned}$$

where $|\tilde{u_R}\rangle =\sigma _z|u_R\rangle ,|\tilde{u_L}\rangle =\sigma _z|u_L\rangle$ which follow the condition $\langle u_L|u_R\rangle =\langle \tilde{u_L}|\tilde{u_R}\rangle =1,\langle u_L|\tilde{u_R}\rangle =\langle \tilde{u_L}|u_R\rangle =0$ leading to the Q-matrix $Q(\beta )=|\tilde{u_R}(\beta )\rangle \langle \tilde{u_L}(\beta )|-|u_R(\beta )\rangle \langle u_L(\beta )|$. The topological invariant in generalized Brillouin zone is given by

60 $$\begin{aligned} W=\oint \frac{dk}{4\pi i}Tr\left[ \sigma _zH^{-1}(k)\frac{\partial }{\partial k}H(k)\right] \end{aligned}$$

where *H*(*k*) can be written in the corrected form as

61 $$\begin{aligned} H(k-ilnr_0)=\left( \begin{matrix} 0&{}&{}t+\delta +t^{\prime }r_0^{-1}e^{-ik}\\ t-\delta +t^{\prime }r_0e^{ik}&{}&{}0 \end{matrix}\right) , \end{aligned}$$

which defines the topological invariant in the generalized Brillouin zone^10,85–88^.

*Extended-range couplings* For the extended-range models such as $r=2$, the real space wavefunctions can be written as,

62 $$\begin{aligned} \frac{t^{\prime }}{2^{\alpha }}\psi _{n-2,B}+ t^{\prime }\psi _{n-1,B}+(t+\delta )\psi _{n,B}= & {} E\psi _{n,A}\nonumber \\ \frac{t^{\prime }}{2^{\alpha }}\psi _{n+2,B}+ t^{\prime }\psi _{n+1,A}+(t-\delta )\psi _{n,A}= & {} E\psi _{n,B} \end{aligned}$$

with energy relation

63 $$\begin{aligned} ((t+\delta )+t^{\prime }\beta ^{-1}+\frac{t^{\prime }}{2^{\alpha }}\beta ^{-2}) ((t-\delta )+t^{\prime }\beta +\frac{t^{\prime }}{2^{\alpha }}\beta ^2)=E^2 \end{aligned}$$

Since, the energy equation takes quartic form, there occurs four roots with

64 $$\begin{aligned} \beta _{1,2,3,4}=\frac{-t^{\prime }\pm \sqrt{(t^{\prime })^2-4(t+\delta )\frac{t^{\prime }}{2^{\alpha }}}}{2(t+\delta )},\frac{-t^{\prime }\pm \sqrt{(t^{\prime })^2-4(t-\delta )\frac{t^{\prime }}{2^{\alpha }}}}{2\frac{t^{\prime }}{2^{\alpha }}}. \end{aligned}$$

The topological invariant can be calculated by using Eq. (60), where

65 $$\begin{aligned} H(k-ilnr_0)=\left( \begin{matrix} 0&{}&{}t+\delta +t^{\prime }r_0^{-1}e^{-ik}+\frac{t^{\prime }}{2^{\alpha }}r_0^{-2}e^{-2ik}\\ t-\delta +t^{\prime }r_0e^{ik}+\frac{t^{\prime }}{2^{\alpha }}r_0^{2}e^{2ik}&{}&{}0 \end{matrix}\right) , \end{aligned}$$

where $\beta$ is given by Eq. (64). The construction of generalized Brillouin zone is highly sensitive towards the system parameters and there can occur the problems of calculation accuracy^10,86–90^. This construction can be unreliable due to numerical diagonalization errors and sensitivity towards matrix dimensions^89^. Thus it can be really difficult and time consuming to construct the generalize Brillouin zone for extended models to verify the edge mode behavior. However, we observe some efforts to define topological invariant in generalized Brillouin zone for various extended models^10,77,86^, which is similar to our model. Here we limit our study to the second interacting neighbor where the Uhlmann phase at finite temperature shows its limitation in recognizing the higher topological invariant.

### Derivation of Uhlmann geometric phase

The parallelism condition is explained by the H$\ddot{u}$bner relation,

66 $$\begin{aligned} A(\partial _k\psi )=\sum _{ij}|u_i\rangle \frac{\langle u_i|\left[ \partial _k\psi ,\psi \right] |u_j\rangle }{p_i+p_j}\langle u_j|, \end{aligned}$$

where $\psi =\sqrt{\rho },p_i$ and $|u_i\rangle$ are the eigenvector and eigenvalues of $\rho$ respectively. For a Bloch sphere (of two level system), the above relation becomes

67 $$\begin{aligned} A(\partial _k\psi )= & {} (\sqrt{p_1}-\sqrt{p_2})^2 (|u_1\rangle \langle u_1|\partial _ku_2\rangle \langle u_2|+|u_2\rangle \langle u_2|\partial _ku_1\rangle \langle u_1|), \end{aligned}$$

with

$$\begin{aligned} p_1= & {} \frac{1}{2}\left( 1+\tanh \left( \frac{\Delta _k}{2T}\right) \right) ,p_2=\frac{1}{2}\left( 1-\tanh \left( \frac{\Delta _k}{2T}\right) \right) ,\\{} & {} \quad |u_1\rangle =\frac{1}{\sqrt{2}}\left( \begin{matrix} 1\\ e^{i\theta } \end{matrix}\right) , |u_2\rangle =\frac{1}{\sqrt{2}}\left( \begin{matrix} 1\\ -e^{i\theta } \end{matrix}\right) . \end{aligned}$$

For the Abelian condition, Eq. (67) becomes

68 $$\begin{aligned} A(\partial _k\psi )=\frac{i}{2}(\partial _k\theta )(\sqrt{p_1}-\sqrt{p_2})^2\left( \begin{matrix} -1&{}&{}0\\ 0&{}&{}1\\ \end{matrix}\right) . \end{aligned}$$

Over the closed loop, the group element becomes

69 $$\begin{aligned} U(k)=exp(\oint dk(\partial _k\psi ))=\left( \begin{matrix} e^{iB}&{}&{}0\\ 0&{}&{}e^{-iB}\\ \end{matrix}\right) , \end{aligned}$$

with $B=\frac{1}{2}\oint dk(\partial _k\psi )(\sqrt{p_1}-\sqrt{p_2})^2$. It is to be noted that, the term *B* need not be periodic, even though $\theta _k$ is periodic. Uhlmann phase is given by the phase factor of Eq. (69).

70 $$\begin{aligned}{} & {} Tr[\psi _{||}^{\dagger }(k)\psi _{||}(k)]= & {} \frac{1}{2}(\sqrt{p_1(0)}+\sqrt{p_2(0)})(\sqrt{p_1(k)}+\sqrt{p_2(k)})\cos (k)+\frac{1}{2}(\sqrt{p_1(0)}-\sqrt{p_2(0)})(\sqrt{p_1(k)}-\sqrt{p_2(k)})\cos (\phi +B),\nonumber \end{aligned}$$

where $\psi _{||}(k)=\psi (k)U(k)$. For $\phi (0)=0$, we get

71 $$\begin{aligned} Tr[\psi _{||}^{\dagger }(k)\psi _{||}(k)]= & {} \cos (B) =\cos \left( \frac{1}{2}\oint dk\left( \partial _k\theta )(1-sech\left( \frac{\Delta _k}{2T} \right) \right) \right) . \end{aligned}$$

With the limit $T\rightarrow 0$, the system drives towards pure state limit and Eq. (71) becomes

72 $$\begin{aligned} \textrm{Tr}[\psi _{||}^{\dagger }(k)\psi _{||}(k)]= & {} \cos \left( \frac{1}{2}\oint dk\left( \partial _k\theta \right) \right) , \end{aligned}$$

with Uhlmann phase^31,60^

73 $$\begin{aligned} W_U= & {} Arg\left[ Tr[\psi _{||}^{\dagger }(k)\psi _{||}(k)]\right] =Arg\left[ \cos \left( \frac{1}{2}\oint dk\left( \partial _k\theta \right) \right) \right] . \end{aligned}$$

Similar methodology can be adopted to define the geometric phase for open boundary condition. Here the term *B* (Eq. 69) behaves independent of periodicity, which signals the generalization of Uhlmann phase to incorporate non-Hermitian skin effect. By replacing the Block wave function $u_k$ with non-Block function $\beta$, this can be achieved. The parallelism of the density matrix allows the wavefunctions to define Uhlmann phase at finite temperature. The ‘Q’ matrix defines the parallel condition and the trace of the matrix defined the geometric phase in the generalized Brillouin zone.

## Data Availability

The datasets used and/or analyzed during the current study available from the corresponding author on reasonable request.
